# In Vivo Anticonvulsant Activity of Extracts and Protopine from the *Fumaria schleicheri* Herb

**DOI:** 10.3390/scipharm84030547

**Published:** 2015-12-06

**Authors:** Yuliya Prokopenko, Vadim Tsyvunin, Sergey Shtrygol’, Victoriya Georgiyants

**Affiliations:** 1 Department of Drugs Quality, Standardization and Certification, Institute of Pharmacy Professionals Qualification Improvement, National University of Pharmacy, Pushkinska 53, Kharkiv 61002, Ukraine; 2 Department of Pharmacology, National University of Pharmacy, Pushkinska 53, Kharkiv 61002, Ukraine; shtrygol@mail.ru; 3 Department of Pharmaceutical Chemistry, National University of Pharmacy, Pushkinska 53, Kharkiv 61002, Ukraine; vgeor@ukr.net

**Keywords:** *Fumaria schleicheri*, anticonvulsant activity, flavonoid fraction, alkaloid fraction, protopine, protein-polysaccharide fraction

## Abstract

The present study aimed to investigate the role of several biologically active compounds from *Fumaria schleicheri* Soy.-Will. in anticonvulsant models. The flavonoid fraction, alkaloid fraction, individual alkaloid protopine, and polysaccharide-protein complex were isolated from the *Fumaria schleicheri* herb and studied along with *Fumaria schleicheri* dry extract in mice with pentylenetetrazole-induced seizures. According to empirical results, the expressed anticonvulsant effect of *Fumaria schleicheri* dry extract depends on the synergism of biologically active compounds in herbal medicine, although some individual substances (mostly protopine and the protein-polysaccharide fraction) have shown moderate anti-seizure activity.

## 1. Introduction

Epilepsy is a disorder of the brain characterised by an enduring predisposition to generate epileptic seizures and thus, is a precursor to serious neurobiological, cognitive, psychological, and social consequences. Despite the availability of a wide range of antiepileptic medicines, the development of new drugs derived from herbal remedies with anticonvulsant properties remains problematic. It is well-known that herbal remedies are inherently safer compared with synthetic therapies, even during long-term use [1,2]. In addition, herbal medicines usually have a complex influence on disease pathogenesis. This property is usually due to their multicomponent composition. The synergism of biologically active compounds in herbal remedies marks the efficiency of their individual substances or fractions [3,4,5].

Previously, the expressed anticonvulsant properties of the dry extract of *Fumaria schleicheri* and other *Fumaria* species, in particular the pharmacopoeial herb *Fumaria officinalis* L., were discovered [6,7,8]. Despite the fact that the *Fumaria schleicheri* herb has not yet been standardised, the study of its chemical composition has been carried out simultaneously with the development of the State Pharmacopoeia of Ukraine monograph “*Fumaria officinalis* L.”. Thus, it was found that *Fumaria schleicheri* and *Fumaria officinalis* herbs have the same qualitative composition of alkaloids, but differ in their total content calculated as protopine [9]. Also, differences were found in the content of flavoniods, amino acids, polyphenols, phenolic acids, and lipophilic compounds [10,11].

In the first stage of the *Fumaria* species anticonvulsant properties study, it was established that among other *Fumariaceae* family members, *Fumaria schleicheri* dry extract has shown a pronounced anticonvulsant activity that is probably due to differences in its chemical compositions [7].

The anticonvulsant properties of *Fumaria schleicheri* dry extract were also confirmed on experimental seizure models with different neurochemical mechanisms, e.g., picrotoxine-induced, tiosemicarbazide-induced, and strychnine-induced seizures, and convulsions induced by camphor. Later, on the model of acute paroxysms induced by maximal electroshock and pentylenetetrazole-induced kindling, the strong antiepileptic potential of *Fumaria schleicheri* dry extract was identified [12,13]. The displayed results formed the basis of the Ukraine patent [14] and after that, demanded more in-depth study of the *Fumaria schleicheri* anticonvulsant properties*.*

While analyzing publications devoted to this given scientific subject, several results were found in the area of studies on the neurotropic mechanisms of action for different groups of substances allocated from medicinal plants [15,16,17,18,19,20].

Considering the obtained results, it was decided to analyze the role of biologically active substances or fractions from *Fumaria schleicheri* in anticonvulsant treatment for a deeper understanding of *Fumaria schleicheri* dry extract mechanisms of anti-seizure activity. This probably aided in identifying new possibilities for the pharmacological correction of epilepsy. The present research was conducted to investigate the anticonvulsant activity of the protein-polysaccharide fraction, alkaloid fraction, flavonoid fraction, and the main alkaloid of Fumaria protopine.

## 2. Materials and Methods

### 2.1. Chemicals

Reagents from Sigma-Aldrich (St. Louis, MO, USA) and Merck (Darmstadt, Germany) were used and prepared according to the requirements of the State Pharmacopoeia of Ukraine and European Pharmacopoeia.

Pentylenetetrazole was purchased from Sigma-Aldrich.

Sodium valproate was used in the form of syrup 57.64 mg/1 mL (trade name Depakine, Sanofi-Aventis, Gentilly, France).

### 2.2. Plant Material

Above-ground parts of *Fumaria schleicheri* were gathered during the flowering season (in full bloom) in Ukraine. Species identification was performed in the Pharmaceutical Botany Department, National University of Pharmacy, Kharkiv, Ukraine. The herbal material was cleaned, washed, and dried. After complete drying, the dry herbs were stored at room temperature. Then, herb samples were powdered and used for further research.

### 2.3. Extraction

The extract was prepared as follows: 100 g of the air-dried and powdered herb of *Fumaria schleicheri* were placed into a percolator, and extraction was allowed to run using water as a solvent in a ratio 1 to 20 at 80 °C for 2 h. Then, the extract was filtered and concentrated in a vacuum-evaporation apparatus at 50–60 °C and at 80–87 kPa to a thick consistency. Finally, the extract was dried under the vacuum in the desiccators to yield 11.28 g of the dry extract with a residual moisture content of 5%.

### 2.4. Flavonoids Assay

The flavonoids assay was carried out according to the State Pharmacopoeia of Ukraine method [21]. The extract was dissolved in ethanol 70%; the ultrasound extraction was carried out for 20 min at room temperature. The obtained solution was filtered, mixed with 3 mL of NaNO_2_ (5% w/v), and after 5 min, 0.5 mL of AlCl_3_ (2% w/v) was added. The mixture was left for 10 min at room temperature and then subjected to spectral analysis in the range of 300–600 nm against the blank, where AlCl_3_ solution was substituted by water.

Flavonoid standard solutions of 100 μM were used.

### 2.5. Alkaloids Assay

The alkaloids assay was carried out according to the State Pharmacopoeia of Ukraine and European Pharmacopoeia method [21,22]. The extract was moistened by dilute ammonia solution. Ethyl acetate was added, and the mixture was shaken for 15 min. The procedure was repeated with another portion of ethyl acetate. Obtained solutions were combined, filtered, and evaporated to dryness. The residue was dissolved by sonication in sulphuric acid of 0.05 M. Then, the pH was adjusted to 9–10 with concentrated ammonia. Ethyl acetate solution was added, and the mixture was shaken. The organic layer was evaporated, and the residue was taken up with anhydrous acetic acid. The obtained test solution was titrated with perchloric acid 0.02 M with potentiometric determination of the end-point.

### 2.6. Protopine Assay

To the dry *Fumaria schleicheri* extract, sulphuric acid was added; the mixture was stirred for 15 min. The obtained solution was filtered. Concentrated ammonia and ethyl acetate were added; the mixture was stirred and then centrifuged. The upper organic layer was dried over anhydrous sodium sulphate and then evaporated to dryness. The residue was taken up with methanol.

Protopine hydrochloride was used as the reference solution.

A preparative thin-layer chromatography silica gel plate was used. The mobile phase for thin-layer chromatography according to the State Pharmacopoeia of Ukraine and European Pharmacopoeia thin-layer chromatography identification method was used [21,22].

The alkaloid zone at the standard substance of the protopine hydrochloride zone level was isolated and eluted. After recrystallisation from methanol, crystals of protopine were obtained. The protopine assay was carried out according to the State Pharmacopoeia of Ukraine and European Pharmacopoeia method [21,22].

### 2.7. Protein-Polysaccharides Fraction Identification and Assay

The protein-polysaccharide fraction of *Fumaria* was extracted according to Huang’s method [23] with a slight modification. The extract was suspended in a triple volume of ethanol (96%); the ultrasound extraction was carried out for 60 min at room temperature. The obtained extract was transferred into centrifuge tubes and centrifuged for 15 min at 2700 rpm. The residue was rinsed with 70% ethanol and vacuum-dried at 60 °C for 20 h. Identification of polysaccharides and proteins was carried out according to Khalid’s method [24]. The protein-polysaccharide fraction assay was carried out by a gravimetric method.

### 2.8. Isolation of Active Compounds

Flavonoids were extracted from the *Fumaria schleicheri* herb with 70% ethanol; ethanol was allowed to evaporate after extraction. The complete quantity of flavonoids from the water residue was extracted by ethyl acetate. Then, the obtained flavonoids were quantified by UV spectrophotometric determination as aluminium chelate complexes, as described in the State Pharmacopoeia of Ukraine [22]. The extraction efficiency was approximately 1.2%.

For alkaloid extraction, the *Fumaria schleicheri* herb was processed by ammonia solution, ethanol (96%), and a diethyl ether mixture. Isoquinoline alkaloids were extracted by diethyl ether until complete exhaustion of the herb (by reaction with Mayer’s reagent). The obtained extract was evaporated to reduce the volume. Next, the extract was processed with 0.05 M sulfuric acid solution and after alkalisation, by diethyl ether. The resulting extract was evaporated to ensure dryness. The extraction efficiency was 0.24%.

For the protopine and protein-polysaccharide fraction extraction, the same methods as described above were used. The extraction efficiency of protopine was 0.08%; for the protein-polysaccharide fraction, the extraction efficiency was 24%.

### 2.9. Animals

Adult random-bred albino mice of either sex weighing 22–28 g were included in the present study. The animals were obtained from the vivarium of the Central Scientific-Research Laboratory, the National University of Pharmacy, Kharkiv, Ukraine, and maintained at 19–24 °C and at 50% humidity in a well-ventilated room with a 12h light/dark cycle. Mice were housed in standard polypropylene cages with free access to food (“Mouse diet”, LabDiet, St. Louis, MO, USA) and water. However, food—not water—was withdrawn 12 h before and during the experiment. All the experimental protocols were approved by the Bioethics Commission of the National University of Pharmacy (protocol No. 2, February 17, 2016). Experiments were conducted in accordance with “Directive 2010/63/EU of the European Parliament and of the Council of 22 September 2010 on the protection of animals used for scientific purposes”. 

### 2.10. Acute Toxicity Studies

The dry extract of *Fumaria schleicheri* was dissolved in distilled water and administered to six mice at a dose of 5000 mg/kg intragastrically. After 14 days, no animal deaths were observed.

### 2.11. Pentylenetetrazole-Induced Seizures

A total of 91 mice were used in the present study. The animals were randomly divided into 11 groups of 6–10 mice:

Group I: Control (treated with only distilled water at a dose of 0.1 mL/10 g)
Group II: treated with a dry extract of *Fumaria schleicheri* (100 mg/kg)
Group III: treated with a flavonoid fraction (0.05 mg/kg)
Group IV: treated with a flavonoid fraction (0.5 mg/kg)
Group V: treated with an alkaloid fraction (0.02 mg/kg)
Group VI: treated with an alkaloid fraction (0.2 mg/kg)
Group VII: treated with protopine (0.005 mg/kg)
Group VIII: treated with protopine (0.05 mg/kg)
Group IX: treated with polysaccharide-protein complex (1.8 mg/kg)
Group X: treated with polysaccharide-protein complex (18 mg/kg)
Group XI: Positive control (treated with sodium valproate at a dose of 300 mg/kg)

In the first case, the selected doses of biologically active substances are equivalent to the amount of the relevant compound in 100 mg of the dry extract of *Fumaria schleicheri*, while in the second case, the dose is increased ten-fold.

All tested preliminary samples were dissolved in distilled water and administrated into the stomach at the calculated doses for 2 days. The control group was treated with distilled water intragastrically in a similar mode. Pentylenetetrazole (80 mg/kg) was administrated subcutaneously on the second day for 30 min after the introduction of the tested samples. Then, the animals were placed into individual transparent plastic boxes and observed for 1 h for clonic-tonic seizures.

Assessment of anticonvulsant action was conducted according to time (latency period, duration of the convulsive period, and time of death), conventional indicators (number of clonic-tonic convulsions in one mouse, severity of seizures), and alternative indicators (% of mice with clonic and tonic convulsions, lethality).

If convulsions had not occurred for 1 h, then the latency period would equal 60 min. The severity of seizures was evaluated according to a scale ranging from 1 to 6: 1—trembling; 2—circus movement; 3—clonic seizures; 4—clonic-tonic seizures with a lateral position; 5—tonic extension; 6—tonic extension leading to the animal’s death.

### 2.12. Statistical Analysis

Results are expressed as the mean ± standard error of mean (SEM). Statistical differences between groups were analyzed using Student’s t-test (in the case of normal distribution), the Mann-Whitney U test, and the Fisher angular transformation (with Yates correction, if necessary). The level of statistical significance was considered as *p* < 0.05.

## 3. Results and Discussion

The content of alkaloids, flavonoids, protein-polysaccharide fraction, and protopine in *Fumaria schleiheri* dry extract was studied and recalculated per 100 mg of extract. The results are summarised in Table 1.

In mice subjected to pentylenetetrazole-induced seizures [25], the *Fumaria schleicheri* dry extract as the reference drug sodium valproate has shown potent anticonvulsant activity, resulting in a significant increase in the latency period of the appearance of the first seizure and reducing the duration of the convulsive period in the group (Table 2). The isoquinoline alkaloid protopine, at a dose of 0.005 mg/kg, rose the latency period in comparison with the control group with statistical significance. This effect is dose-dependent and is confirmed by the absence of reliable differences within a certain time frame in the control group and the group of animals that received protopine at a dose of 0.05 mg/kg. The increase in the latency period, while taking the flavonoid fraction at doses of 0.05 and 0.5 mg/kg, did not exhibit statistical significance. Finally, other compounds did not have an influence on the time indicators of the experimental seizures.

The results of the evaluation of the conventional indicators of pentylenetetrazole-induced seizures are presented in Table 3.

The flavonoid fraction at the low dose, the alkaloid fraction at the high dose, the protopine at the low dose, and the protein-polysaccharide fraction at both doses as the *Fumaria schleicheri* dry extract at a dose of 100 mg/kg reduced the number of clonic-tonic convulsions in one mouse in the experimental groups with statistical significance. In addition, the protein-polysaccharide fraction at a dose of 18 mg/kg and *Fumaria schleicheri* dry extract led to a decrease in the severity of seizures in groups, indicating the observable anticonvulsant activity of these drugs. Sodium valproate, similar to the *Fumaria schleicheri* dry extract, reduced the number of clonic-tonic convulsions in one mouse and the severity of seizures.

Alternative indicators of experimental convulsions are shown in Table 4.

The dry extract of *Fumaria schleicheri* and the reference drug sodium valproate in experimental doses had a pronounced anti-seizure effect based on the reduction in the number of mice with clonic and tonic convulsions and the decreasing lethality in the group compared with the control animals. The flavonoid fraction (0.05 mg/kg), protopine (0.005 mg/kg), and the protein-polysaccharide fraction (18 mg/kg) reduced the tonic (but not clonic) phase of experimental seizures. Also, the protein-polysaccharide fraction at a dose of 18 mg/kg decreased the animals’ lethality in the group with statistical significance. The flavonoid fraction, alkaloid fraction, individual alkaloid protopine, and protein-polysaccharide fraction from *Fumaria schleicheri* herb, even at a ten-fold increased dose separately, did not show the potent anticonvulsant activity, unlike *Fumaria schleicheri* dry extract. It is likely that their effect depends on their impact on different pathways of seizures. It is well-known that natural flavonoids have been shown to possess a selective and relatively mild affinity for the benzodiazepine-binding site of the GABA-barbiturate-benzodiazepine receptor complex and have a pharmacological profile compatible with a partial agonistic action. Isoquinoline alkaloids (in particular, protopine) most probably provide the suppression of glutamate excitotoxicity by inhibiting the Ca^2+^ influx. Polysaccharides from the protein-polysaccharide fraction can inhibit seizures through mechanisms that involve the inhibition of ionotropic glutamate receptors and the interleukin-1β pathway. The protein component of the fraction can serve as a substrate for the synthesis of neuroactive amino acids in the central nervous system.

The dry extract of *Fumaria schleicheri* has shown expressed anticonvulsant properties that probably depend on the synergism of flavonoids, alkaloids, and protein-polysaccharide fraction influences. According to the strength of the effect, the total extraction medication was not inferior to the classical anticonvulsant sodium valproate.

## 4. Conclusions

The anticonvulsant activity of biologically active substances from *Fumaria schleicheri* has been reported for the first time. It was established that the potent anti-seizure effect of *Fumaria schleicheri* dry extract depends on the synergistic combination of different compounds, but the main mechanism of this anticonvulsant effect probably stems from the isoquinoline alkaloid protopine and protein-polysaccharide fraction content, which have shown moderate anticonvulsant activity.

## Figures and Tables

**Table 1:** Content of flavonoids, alkaloids, protopine, and protein-polysaccharide fraction in *Fumaria schleicheri* dry extract.

Compound	Content (mg), per 100 mg
Flavonoids	0.05
Isoquinoline alkaloids	0.02
Protopine	0.005
Protein-polysaccharide fraction	1.8

**Table 2:** Estimation of time indicators of experimental pentylenetetrazole-induced seizures in mice.

Group	*n*	Dose, mg/kg	Latency Period, min	Duration of the Convulsive Period, min	Time of Death, min
Control	10	-	2.63 ± 0.73	11.51 ± 3.41	11.37 ± 1.74
*Fumaria schleicheri* dry extract	8	100	19.39 ± 8.97 *	3.83 ± 1.91 *	12.97 ± 0.94
Flavonoid fraction	10	0.05	4.30 ± 1.43	7.99 ± 2.08	8.95 ± 1.41
	8	0.5	4.93 ± 1.63	10.60 ± 2.39	13.58 ± 2.05
Alkaloid fraction	8	0.02	2.89 ± 0.29	11.89 ± 1.58	13.32 ± 1.48
	8	0.2	3.38 ± 0.44	9.56 ± 3.00	12.94 ± 2.97
Protopine	8	0.005	4.43 ± 0.85 *	11.15 ± 1.67	15.74 ± 1.70
	7	0.05	3.64 ± 0.60	8.63 ± 1.85	11.82 ± 1.93
Protein-polysaccharide fraction	8	1.8	2.87 ± 0.55	6.05 ± 1.60	8.10 ± 1.58
	10	18	3.69 ± 0.87	7.61 ± 1.31	13.27 ± 3.36
Sodium valproate	6	300	11.05 ± 1.97 *	4.14 ± 1.84 *	9.15 ± 4.52

Results are means ± SEM. * Significant at *p* < 0.05 compared with the control group.

**Table 3:** Estimation of conventional indicators of experimental pentylenetetrazole-induced seizures.

Group	*n*	Dose, mg/kg	Number of Clonic-Tonic Convulsions in 1 Mouse	Severity of Seizures, Points
Control	10	-	4.80 ± 0.84	5.80 ± 0.20
Dry extract of *Fumaria schleicheri*	8	100	1.13 ± 0.30 *	3.38 ± 0.89 *
Flavonoid fraction	10	0.05	2.60 ± 0.45 *	4.80 ± 0.49
	8	0.5	3.50 ± 0.68	5.63 ± 0.38
Alkaloid fraction	8	0.02	3.50 ± 0.27	5.50 ± 0.38
	8	0.2	2.38 ± 0.42 *	6.00 ± 0.00
Protopine	8	0.005	2.38 ± 0.38 *	5.25 ± 0.49
	7	0.05	2.86 ± 0.40	5.14 ± 0.46
Protein-polysaccharide fraction	8	1.8	2.38 ± 0.38 *	5.25 ± 0.37
	10	18	2.70 ± 0.37 *	4.00 ± 0.45 *
Sodium valproate	6	300	1.40 ± 0.25 *	3.00 ± 1.10 *

Results are means ± SEM. * Significant at *p* < 0.05 compared with the control group.

**Table 4:** Estimation of alternative indicators of experimental pentylenetetrazole-induced seizures.

Groups	*n*	Dose, mg/kg	% of Mice with Convulsions		Lethality, %
			Clonic	Tonic	
Control	10	-	100	100	90
*Fumaria schleicheri* dry extract	8	100	75 *	37.5 *	37.5 *
Flavonoid fraction	10	0.05	100	60 *	60
	8	0.5	100	87.5	87.5
Alkaloid fraction	8	0.02	100	87.5	75
	8	0.2	100	100	100
Protopine	8	0.005	100	75 *	75
	7	0.05	100	85.7	57.1
Protein-polysaccharide fraction	8	1.8	100	100	75
	10	18	100	40 *	40 *
Sodium valproate	6	300	66.7 *	33.3 *	33.3 *

* Significant at *p* < 0.05 compared with the control group.
